# Exposure to Tebuconazole Drives Cross-Resistance to Clinical Triazoles in *Aspergillus fumigatus*

**DOI:** 10.1021/acsomega.5c07812

**Published:** 2025-10-18

**Authors:** Leandra Martins Meireles, Fabiano Silva Soares, Gonçalo Apolinário de Souza Filho, Rodrigo Scherer

**Affiliations:** † Pharmaceutical Sciences Graduate Program, 125100 Universidade Vila Velha, Espírito Santo 29102-920, Brazil; ‡ Universidade Estadual Norte Fluminense Darcy Ribeiro, Rio de Janeiro 28013-602, Brasil

## Abstract

Multiazole-resistant *Aspergillus fumigatus* threatens antifungal therapy. This study investigated the impact of prolonged exposure to the agricultural triazole tebuconazole (TEB) on the susceptibility of *A. fumigatus* to clinical triazoles and its resistance mechanisms. After 28 days, resistant strains emerged with substantial increases in minimum inhibitory concentrations (MICs): up to 128× for itraconazole, 64× for posaconazole, and 16× for voriconazole. In the absence of TEB, resistance was maintained and associated with maintenance of CYP51A activity, with stable ergosterol levels and efflux pump activity. Among the 193 upregulated proteins identified in proteomics, 14 were related to resistance, 5 to the ergosterol biosynthesis pathway, 7 to the efflux pump activity, and 2 to the heat shock response pathway. These results demonstrate that agricultural triazoles can induce stable and multidrug-resistant phenotypes, emphasizing the need for surveillance of antifungal resistance in environmental and clinical settings.

## Graphical abstract

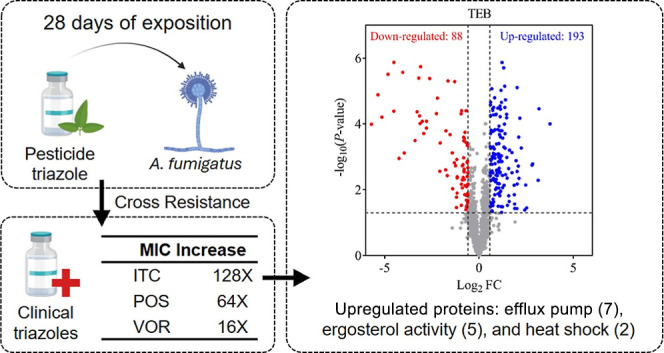

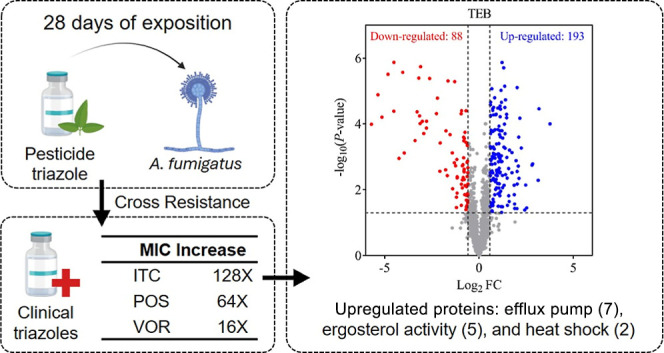

## Introduction

The application of triazole compounds in agriculture is widely used to inhibit the growth of phytopathogens. The need to control phytopathogens is due to the risk of compromising agricultural quality and productivity. Considering the accelerated population growth, inadequate management of agricultural productivity can result in significant losses in the food chain.

However, the continued use of triazoles in the environment has been associated with risks to human health. Research has associated the indiscriminate use of agricultural triazoles with the impairment of antifungal therapies with triazoles, since both have important chemical structural similarity and mechanism of action, favoring the development of cross-resistance.^1–3^

Triazoles block the production of ergosterol, an important sterol present in the fungal cell membrane. Inhibition occurs through interaction with the enzyme lanosterol 14-α-demethylase. This interaction promotes the formation of a sterol considered toxic to the fungal cell membrane, which accumulates and leads to a loss of fluidity and stability, promoting a fungistatic effect on the microorganism’s metabolism.^1–3^

*Aspergillus* fungi are considered human pathogens that are widely distributed in the environment. In this context, they can adapt to the pressure generated by triazoles and develop resistance to available antifungal treatments.^4^ The inhibitory capacity of triazole compounds can be reduced due to molecular phenomena that promote the adaptation of the microorganism to the triazole compound.

Among the known mechanisms are the reduction of the affinity of the target enzyme due to point mutations and the overexpression of the target enzyme. In addition, studies also suggest the participation of efflux pumps (EPs) in the reduction of the inhibitory capacity of triazole compounds.^5–7^ Prolonged contact of microorganisms with triazole compounds can favor adaptation, and studies indicate the occurrence of this phenomenon in both the agricultural and clinical environments.^8,9^

Proteomics makes it possible to identify and quantify proteins altered after exposure of the microorganism to compounds that are stressful to its metabolism. In this way, it is possible to identify active pathways that favor adaptation and changes in gene expression in important metabolic pathways that are directly related to resistance, such as ergosterol biosynthesis, EPs, and stress response. The data generated by this technique are essential to complement genomic and transcriptomic data, providing insight into active resistance mechanisms.^10,11^

Considering that triazole compounds are the therapy of choice for the treatment of clinical infections and the risks of cross-resistance development between clinical and environmental triazoles, this study aims to investigate the risk of resistance development after prolonged in vitro exposure of *A. fumigatus* strains to the agricultural triazole tebuconazole (TEB). In addition, it aims to identify the cellular mechanisms involved in the development of resistance. Elucidation of resistance mechanisms is essential for understanding the risks associated with the indiscriminate use of triazoles in agriculture and its implications for public health.

## Results

The epidemiological cutoff values for clinical triazoles against *A. fumigatus* are described in the literature and serve to identify strain resistance. Strains with MIC below or equal 1.0 μg/mL for ITZ and VCZ, and 0.125 μg/mL for POS, are considered susceptible to clinical triazoles.^12,13^ In the present study, before exposure to TEB (naïve phase), *A. fumigatus* strains presented MIC within the cutoff values (Table 1).

**Table 1:** Minimum Inhibitory Concentration for *A. fumigatus* before (naïve) and after Exposure to Tebuconazole (Adapted)^a^

	MIC (μg/mL)							
	ITZ		VCZ		POS		TEB	
A. fumigatus	naïve	adapted	naïve	adapted	naïve	adapted	naïve	adapted
ATCC	0.125	≥16 [128×]	0.125	2.0 [16×]	0.0625	1.0 [16×]	1.0	4.0 [4×]
AF 293	0.50	≥16 [32×]	1.0	2.0 [2×]	0.0625	4.0 [64×]	4.0	8.0 [2×]
WT 35	0.125	≥16 [128×]	0.25	1.0 [4×]	0.0625	1.0 [16×]	1.0	2.0 [2×]
CEA 17	0.125	≥16 [128×]	0.25	1.0 [4×]	0.0625	0.5 [8×]	1.0	2.0 [2x]
A. flavus								
204304	0.25	0.5 [2×]	0.5	1.0 [2×]	0.0625	0.5 [8×]	1.0	2.0 [2×]

a *naïve*: MIC before exposure to the pesticide; TEB: tebuconazole; ITZ: itraconazole; VCZ: voriconazole; POS: posaconazole; adapted: MIC after exposure to TEB; values in brackets indicate the increase in concentration in relation to the period *naïve*.

### Cross-Resistance to Medical Triazoles in *Aspergillus fumigatus* Induced by Pesticide Tebuconazole

*A fumigatus* strains exposed to TEB adapted to the stress caused by exposure and maintained their physiological activity, without compromising ergosterol production when compared to the control (Figure 1). As a result, the microorganisms showed growth at concentrations 8 times higher than the initial concentration of exposure to TEB (25% MIC TEB). Exposure to TEB also favored the adaptation of the microorganism to clinical triazoles, with significant increases in MIC. An increase of up to 128 times was observed for itraconazole (ITZ), 16 times for voriconazole (VCZ), and 64 times for posaconazole (POS) (Table 1, adapted). In addition, up to 4 times, the TEB MIC was required to inhibit the growth of the microorganism (Table 1, adapted).

**Figure 1:**
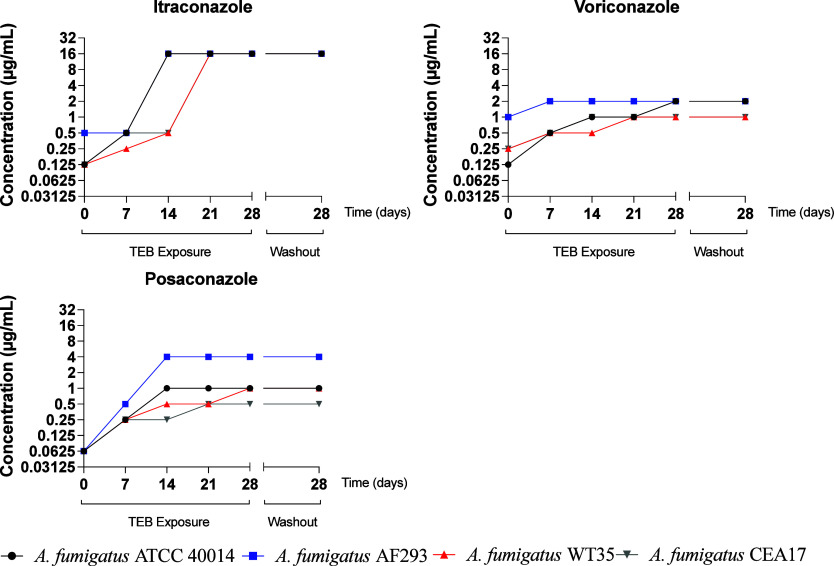
MIC for clinical triazole over time of exposure to TEB.

Exposure to the triazole pesticide TEB induced cross-resistance to clinical triazoles. *A. fumigatus* strains studied were considered resistant to all clinical triazoles after 28 days of exposure to TEB, except for *A. fumigatus* WT 35 and CEA 17 strains for VCZ (1.0 μg/mL). Table 1 presents the MIC values for the triazole compounds before (naïve) and after exposure to TEB for 28 days (adapted).

To determine the exposure time required for *A. fumigatus* strains exposed to triazole pesticides to develop cross-resistance to clinical triazoles, a susceptibility test was performed over 28 days of exposure to TEB. An increase in MIC was observed at 7 days of exposure for all clinical triazoles, with the emergence of resistant strains between 7 and 14 days for ITZ, VCZ, and POS. The resistance profile of the strains did not change after 28 days of growth in the absence of TEB, indicating that the changes in MIC remained stable, thus sustaining resistance (Figure 1).

These results demonstrate the ability of the microorganism to develop tolerance to high concentrations of pesticide TEB and clinical triazoles after a short period of exposure. Tolerance led to a reduction in the inhibitory capacity of these compounds, resulting in the development of resistance to clinical triazoles, which are essential for medical treatment. The data suggest that resistance mechanisms are active in the cell, bypassing the interference of triazole compounds in ergosterol production.

### Triazole Exposure Fails to Disrupt Ergosterol Biosynthetic Pathways in *A. fumigatus*

The decreased inhibitory capacity of triazole compounds in resistant strains may be associated with several mechanisms, including a reduced affinity and increased expression of the triazole target enzyme. In these cases, when the microorganism is exposed to triazole, the ergosterol production pathway remains active. Therefore, an ergosterol quantification assay was performed to investigate the possible contribution of some of these mechanisms in the resistance observed after exposure to TEB. As shown in this study, *A. fumigatus* strains continued to grow at high concentrations of TEB and did not present significant differences in ergosterol levels compared to the control (Figure 2), suggesting that resistance mechanisms are operating in the fungal cell.

**Figure 2:**
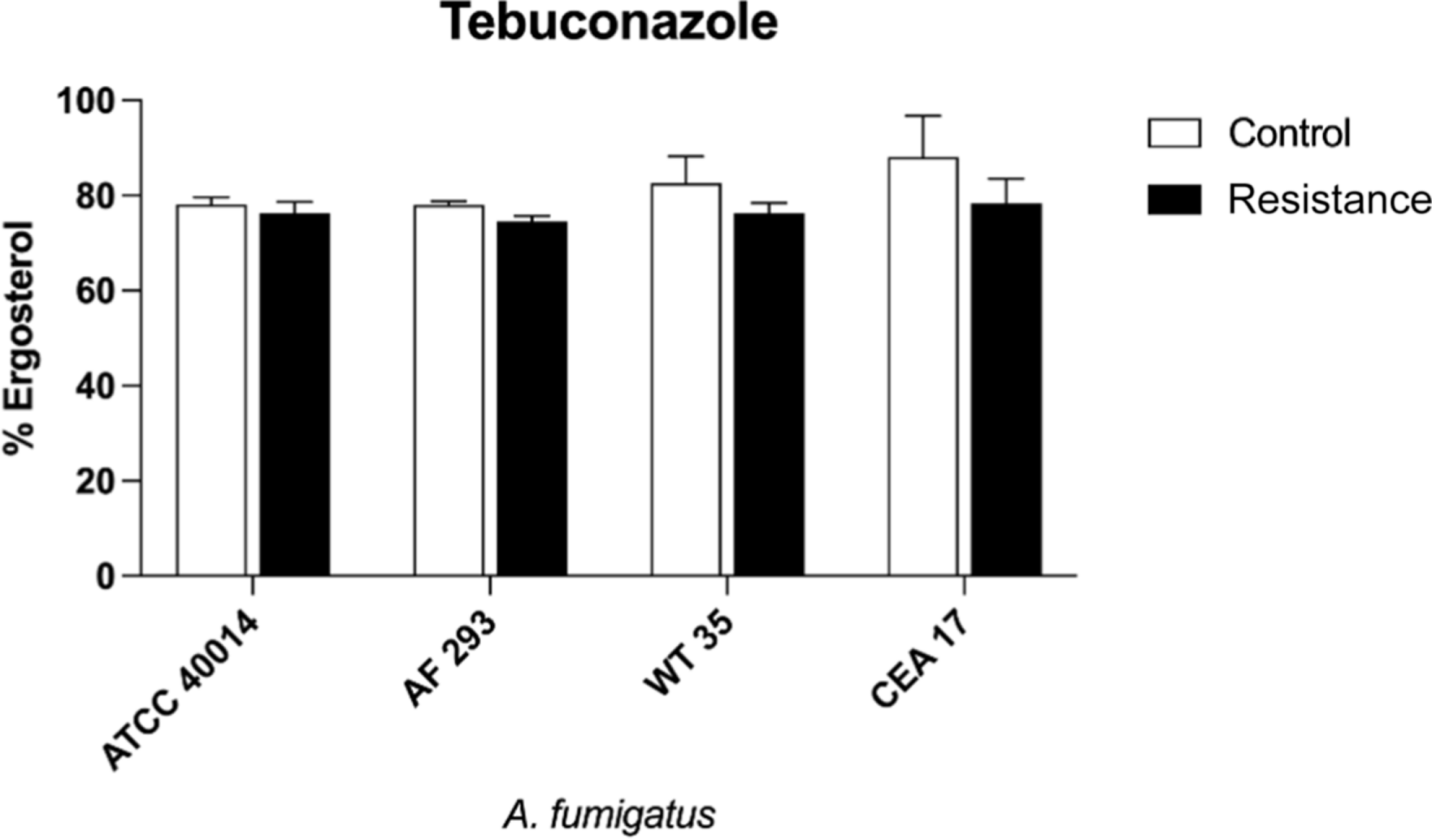
Quantification of ergosterol for *A. fumigatus* strains before and after exposure to TEB.

### Efflux Pump-Mediated Modulation of Drug Resistance

The action of EPs is a mechanism that may contribute to resistance because it facilitates the intracellular elimination of triazole compounds. Thus, the contribution of EP in reducing the inhibitory capacity of triazoles was evaluated by combining the clinical triazoles ITZ, VCZ, and POS with an EP inhibitor.

Adapted *A. fumigatus* strains showed a reduction in the MIC for all clinical triazoles when combined with the EP inhibitor. This combination was considered synergistic, as it resulted in a FICI ≤ 0.5 (Table 2). This result was characterized by a reduction in the MIC for VCZ in <4× for *A. fumigatus* ATCC 40014 and *A. fumigatus* AF293, < 8× for *A. fumigatus* WT 35, and <16× for *A. fumigatus* CEA 17. The reduction in the MIC classified the strains as being sensitive to VCZ. The results obtained in this test suggest the participation of EP in the observed resistance phenotype.

**Table 2:** Fractional Inhibitory Concentration Index (FICI) after Exposure to TEB^a^

A. fumigatus	ITZ	CCCP	ITZ/CCCP	FICI	interpretation
ATCC40014	16.0	16.0	2.0/0.25	0.1	synergistic
AF 293	16.0	16.0	8.0/0.5	0.5	synergistic
WT35	16.0	8.0	0.125/2.0	0.2	synergistic
CEA 17	16.0	16.0	4.0/0.25	0.3	synergistic
A. fumigatus	VCZ	CCCP	VCZ/CCCP	FICI	interpretation
ATCC40014	2.0	4.0	0.5/1.0	0.5	synergistic
AF 293	2.0	4.0	0.5/1.0	0.5	synergistic
WT35	1.0	1.0	0.125/0.25	0.4	synergistic
CEA 17	1.0	1.0	0,0625/0.125	0.2	synergistic
A. fumigatus	POS	CCCP	POS/CCCP	FICI	interpretation
ATCC40014	1.0	8.0	0.25/2.0	0.5	synergistic
AF 293	2.0	2.0	0.5/0.5	0.5	synergistic
WT35	0.5	4.0	0.125/1.0	0.5	synergistic
CEA 17	1.0	4.0	0.25/0.5	0.4	synergistic

a TEB: tebuconazole; ITZ: itraconazole; VCZ: voriconazole; POS: posaconazole; CCCP: carbonylcyanide *m*-chlorophenylhydrazone; FICI: fractional inhibitory concentration index.

For ITZ, the reduction in the MIC was up to 64-fold. However, despite the increase in MIC, some strains enhanced with the ITZ-resistant phenotype (MIC >1 μg/mL), such as *A. fumigatus* ATCC 40014 (MIC 2.0 μg/mL), *A. fumigatus* AF293 (MIC 8.0 μg/mL), and *A. fumigatus* CEA 17 (MIC 4.0 μg/mL) (Table 2).

For POS, the combination with inhibitors impaired the concentration required to inhibit growth by less than 4-fold for all strains. However, *A. fumigatus* ATCC 40014 (0.25 μg/mL), *A. fumigatus* AF293 (MIC 0.5 μg/mL), and *A. fumigatus* CEA 17 (MIC 0.25 μg/mL) maintained the resistant phenotype.

Considering that the ergosterol level in the adapted strains remained like that observed in the naïve period, and that, even with EP inhibition, some strains still presented MICs above the epidemiological cutoff point defined in the literature, it is possible that the greater expression of the target enzyme or mutations in the enzyme contribute to the observed resistance phenotype.

### Differential Analysis of the Proteome of *A. fumigatus* under Tebuconazole Treatment

For the comparative differential analysis of the *A. fumigatus* proteome, only proteins whose presence or absence was consistent in the three biological replicates of each experimental condition were considered, allowing the identification of proteins exclusive to the treatment or control. Differentially accumulated proteins (DAPs) were defined based on statistically significant differences between the TEB-treated group and the control group, as determined by the *t*-test (*p* < 0.05), in addition to meeting the criteria of variation in the abundance ratio (fold change, FC), being considered positively regulated when FC > 1.5 and negatively regulated when FC < 0.667.

In total, 1327 proteins were identified. Of these, 193 showed upregulation, 88 were downregulated, 7 were exclusive to TEB treatment, 6 were exclusive to the control, and 1015 remained unchanged after exposure to the antifungal agent (Figure 3). Among the upregulated proteins, 5 were associated with the ergosterol biosynthesis pathway, 7 with EP activity, 2 with the heat shock response pathway, 17 with virulence factors, and 2 with proteins with allergenic potential. In turn, among the downregulated proteins, 2 were related to virulence factors, 1 to an allergen, and 3 to the heat shock pathway. Additionally, of the proteins identified exclusively in samples exposed to TEB, 3 were associated with virulence factors.

**Figure 3:**
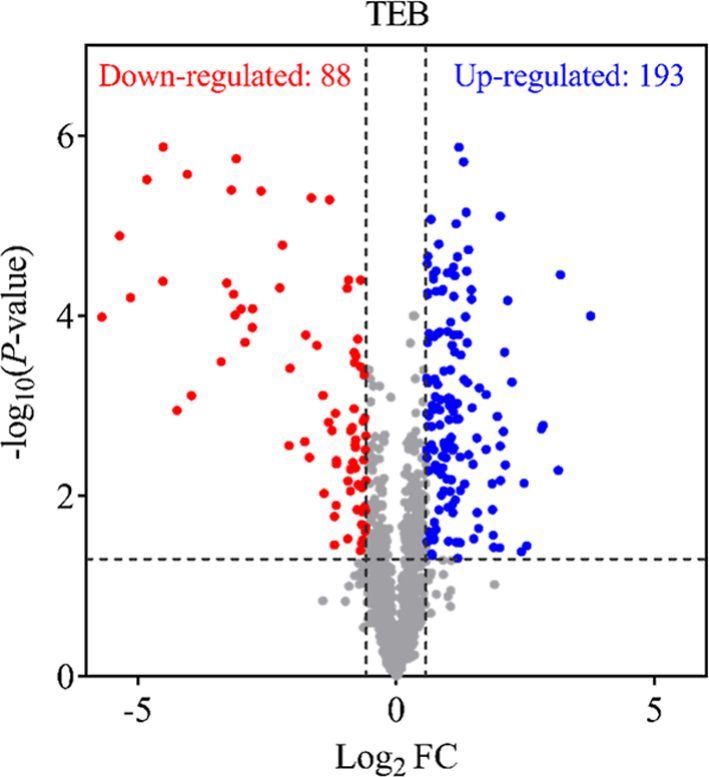
Differentially accumulated proteins in *A. fumigatus* strains after exposure to TEB.

## Discussion

Ergosterol is the main sterol present in the fungal cell membrane and plays an essential role in fungal viability. This compound is essential for maintaining membrane fluidity, in addition to actively participating in protein distribution and cell cycle regulation.^14^ Ergosterol biosynthesis is mediated by a highly conserved pathway, composed of a set of approximately 20 genes, some of which present duplications, such as cyp51A/B, erg24A/B, erg25A/B, erg26A/B, erg3A/B/C, and erg4A/B.^15^

The ergosterol biosynthesis pathway begins with lanosterol, the first precursor sterol, which undergoes methylation at the C-24 position by the action of the enzyme sterol C24-methyltransferase (Erg6A and B), resulting in the formation of eburicol. This compound is then converted to 4,4-dimethylergosta-8,14,24(28)-trien-3β-ol by lanosterol 14-α-demethylase (CYP51A/B). Subsequently, sterol C-14 reductase (Erg24A/B) acts on this intermediate, generating 4,4-dimethylergosta-8.24(28)-dien-3β-ol. The pathway continues with the coordinated action of other enzymes—Erg25A/B, Erg26A/B, and Erg27—culminating in the formation of fecosterol. This, in turn, is converted to episterol through the activity of the enzyme Erg2, which is involved in steroidal isomerization. Episterol is then modified by sterol C-22 desaturase (Erg5), forming ergosta-7,22,24(28)-trien-3β-ol, which is converted to ergosta-5,7,22,24(28)-tetraen-3β-ol by the action of sterol C-5 desaturase (Erg3A/B/C). Finally, the enzyme C-24 reductase (Erg4A/B) catalyzes the final step of the pathway, resulting in the production of ergosterol.^14,16^

Triazole compounds inhibit ergosterol biosynthesis by binding to enzyme CYP51A (lanosterol 14-α-demethylase). This interaction results in the accumulation of eburicol, which, in turn, activates an alternative biosynthetic pathway, in which eburicol is converted to 14-methylfecosterol. This intermediate undergoes the action of the Erg3A/B/C enzymes, generating 14-methylergosta-8.24(28)-dien-3β,6α-diol, a toxic sterol. The accumulation of this sterol in the fungal cell membrane compromises the cellular physiology of *A. fumigatus*, being one of the factors responsible for the antimicrobial activity of azole compounds, which initially exert a fungistatic effect. As described,^17^ azole-sensitive strains first exhibit a fungistatic effect, followed by a mild fungicidal effect. The fungicidal effect of azoles is related to cell wall remodeling, curvature of the cell membrane toward the interior of the fungal cell, compromise of cell wall integrity, loss of cytoplasmic content, fragmentation and mitochondrial lysis, culminating in cell death.^15,17^ The delayed fungicidal effect of triazoles was confirmed after the exposure of *A. fumigatus* to ITZ and VCZ.

Alterations in the ergosterol biosynthesis pathway, induced by exposure to triazole compounds, which result in reduced accumulation of eburicol relative to other sterols, can significantly contribute to the development of resistance.^15^ Several mechanisms of resistance to triazoles are well documented, including amino acid substitution in the active site of CYP51A, which decreases the affinity of the azole for the enzyme, as in the L98H, Y121F, and T289A mutations. In addition, overexpression of the CYP51A enzyme, mutations in the TR34 and TR53 promoter regions, reduced accumulation of triazoles within the fungal cell, mediated by the action of EPs, and activation of stress response pathways, are also important factors in the development of resistance.^15,18^ Furthermore, other resistance pathways have been described, involving the modulation of specific points in the ergosterol biosynthesis pathway. Handelman et al.^19^ reported that overexpression of the cyp51B gene in sensitive *A. fumigatus* resulted in a reduction in triazole susceptibility. Furthermore, overexpression of the cyp51B gene was observed in two triazole-resistant clinical isolates of *A. fumigatus* without the presence of cyp51A mutations. The authors concluded that both mutation and overexpression of the cyp51B gene are associated with triazole resistance.

Hokken et al.^18^ observed that after exposure of A. fumigatus strains to clinical triazoles, ITZ, and isavuconazole, there was an upregulation of several molecular mechanisms, including the overexpression of the cyp51B, erg6, and erg3 genes. Interestingly, the findings of the present study are consistent with the results, since increased expression of the erg3, erg6, and erg24 genes was also observed, which translated into an increase in the amount of proteins expressed after exposure to the triazole TEB. Furthermore, the cyp51B gene also showed high levels of expression in the proteomic analysis. Roundtree et al.^20^ also observed an increase in the expression of the cyp51A and cyp51B genes in strains exposed to VCZ (clinical triazole) and an upregulation of cyp51B after exposure to TEB (agricultural triazole). These findings indicate that exposure to both clinical and agricultural triazoles induces a similar gene expression profile.

In this study, the ergosterol content in the strains before and after exposure to TEB was also analyzed. No significant difference in the ergosterol content was observed between the exposed strains. In a previous study, the ergosterol content was analyzed in wild-type and triazole-resistant strains, with 75.81% ergosterol being found in the wild-type strains, and 84% to 88% in the azole-resistant strains, with no significant difference between the groups.^14^ Additionally, another study analyzed the sterol content in *A. fumigatus* sensitive and resistant to ITZ and VCZ, with TR34/L98H, TR46/Y121F/T289A, and TR53 mutations. It was identified that ergosterol was the main sterol detected, corresponding to 79.5% to 89.5% under basal conditions, 61.8% to 81.1% after exposure to ITZ, and 44.4% to 74.2% after exposure to VCZ. There was no significant difference in ergosterol content between azole-sensitive and azole-resistant isolates.^21^

Azole resistance may occur due to substitutions in the enzyme targeted by azoles, which affect the enzyme’s affinity for the compounds, without altering its activity. In such cases, the sterol composition of the resistant strains remains unchanged throughout the ergosterol biosynthesis pathway. Another hypothesis associated with resistance involves the activity of the EPs. In the present study, a higher expression of proteins belonging to the ABC and MFS families, responsible for the efflux of several compounds, was observed. Natesan et al.^22^ identified resistant isolates that did not present mutations in the cyp51A gene, nor enzyme overexpression related to triazoles. In this context, changes in the expression of resistant multidrug EPs were observed, which significantly contribute to the adaptation of the microorganism to triazole compounds. EPs play a crucial role in the transport of compounds from the intracellular to the extracellular compartment, increasing the efficiency of triazole elimination.^22^ Thus, the triazole cannot effectively bind to the target enzyme or inhibit its function. The increased expression of EPs observed after exposure to TEB may explain the results obtained, reinforcing the hypothesis of cross-resistance in strains exposed to triazole pesticides.

## Materials and Methods

### Microorganism

*A. fumigatus* strains Af 293 (PyrG + ), WT 35, CEA 17 (PyrG + ) (USP- Ribeirão Preto), and ATCC 40014 (INCQS 40182 FIOCRUZ) identified by sequencing the ITS gene of rDNA were used in the study. *Aspergillus flavus* 204304 (UFLA) was included as a test control.

### Susceptibility Testing

The susceptibility testing was performed based on the Clinical and Laboratory Standards Institute (CLSI) M38-A2 protocol.^23^ The clinical triazoles are ITZ, VCZ, POS, and the triazole pesticide TEB (Sigma-Aldrich, St. Louis, USA). The concentrations of triazole compounds ranged from 0.0312 to 16 μg/mL. The inoculum suspension was prepared in saline solution (0.85%) and adjusted in a spectrophotometer to an optical density between 0.09 and 0.13 (530 nm), followed by dilution with RPMI-1640 medium to obtain a density of 0.4 to 5 × 10^4^ CFU/mL. Minimum inhibitory concentration (MIC) was determined after 48 h of incubation at 35 °C. Assays were performed in duplicate. Strains were considered resistant to clinical triazoles when they presented MIC above the epidemiological cutoff values: >1.0 μg/mL for ITZ and VCZ^13^ and >0.125 μg/mL for POS.^12^ Antifungal susceptibility tests were performed according to the CLSI M38-A2 protocol using a reference strain (*A. flavus* 204304) to validate the assay conditions and ensure the reliability of MIC determinations. Negative controls (without inoculum) and positive controls (with inoculum and without pesticide) were also included in all microplates.

### Exposure to Triazole Pesticide

*A. fumigatus* was exposed to increasing concentrations of TEB according to Meireles et al.^24^ Briefly, 1 mL of the spore suspension (0.4 to 5 × 10^4^ CFU/mL) was added to YPD broth containing a subinhibitory concentration of the pesticide TEB in a 1:50 ratio. The initial pesticide concentration was 25% of the MIC described in Table 1 (naïve), on days 0 to 7; half the MIC from days 7 to 14; MIC from days 14 to 21; and 2x MIC from days 21 to 28. The samples were incubated at 35 °C on an orbital shaker (180 rpm). After the exposure period, the microorganism was cultivated in YPD broth for 28 days in the absence of pesticide (adapted) under the same conditions of temperature and agitation.

### Evaluation of Resistance by Exposure to TEB

A susceptibility test was performed to verify the development of clinical triazole resistance after exposure to TEB. For this, on days 7, 14, 21, and 28 of exposure and after 28 days of the adapted period, a sample of the fungal cell mass was removed from the YPD broth with pesticide and grown on PDA. The susceptibility testing was performed based on the CLSI.

### Determination of the FICI

This assay was performed using an inhibitor of EP activity, carbonylcyanide *m*-chlorophenylhydrazone (CCCP).^22^ The determination of FICI was performed by combining each clinical triazole (ITZ, VCZ, and POS) with CCCP. The concentration of clinical triazoles ranged from 0.0312 to 16 μg/mL and CCCP from 0.25 to 16 μg/mL. The assay was performed on *A. fumigatus* strains after 28 days of exposure to TEB. The combination that produced a FICI ≤0.5 indicated synergism in action between the clinical triazoles and inhibitor CCCP. To calculate the FICI, the formula was used: FICI = [MIC clinical triazoles combined/MIC clinical triazoles alone] + [MIC inhibitor combined/inhibitor alone].

### Quantification of Ergosterol

Twenty milligram of fungal cell mass was weighed and submitted to sterol extraction in two stages. The first step consisted of adding 3 mL of 25% ethanolic potassium hydroxide solution with stirring for 1 min on a magnetic stirrer plate. Then the samples were incubated in a water bath at 85 °C for 1 h, followed by cooling to room temperature. In the second step, 1 mL of sterile water and 3 mL of *n*-hexane were added to the samples and stirred for 3 min. The supernatant was removed (200 μL) and added to 800 μL of ethanol for reading in a spectrophotometer at 281.5 and 230 nm. The reading results were used to calculate ergosterol levels, which were expressed as the percentage of ergosterol per wet weight of the fungal cell. Ergosterol and its intermediate 24(28)-DHE (24(28) dihydroergosterol) are absorbed at 281.5 nm. Thus, to calculate the total sterols, the following formula was used: % sterol = [*A*_281.5_/*E* × *F*]/pellet weight, where *F* = ethanol dilution factor and value *E* (in percentage per centimeter) determined by crystalline ergosterol = 290. However, only 24(28)-DHE is absorbed at 230 nm. In this way, the amount of this sterol can be calculated with the following formula: % 24(28)-DHE = [*A*_230_/ *E* × *F*]/pellet weight, where *F* = ethanol dilution factor and value *E* (in percentage per centimeter) determined by 24(28)-DHE = 518. Thus, the amount of ergosterol produced was determined by subtracting the total amount of sterol produced by the amount of 24(28)-DHE: % ergosterol = [% sterol – % 24(28)-DHE]. Statistical analysis was performed using the *t*-test. Values *p* ≤ 0.005 were considered a significant difference.^25,26^

### Proteomic Analyses

#### Protein Extraction and Digestion

*A. fumigatus* (ATCC 40014) was grown for 5 days on a Sabouraud dextrose agar at 37 °C. A suspension containing 10^6^ conidia/mL was added to 50 mL of YPD broth supplemented with 0.5 μg/mL TEB and incubated for 24 h at 37 °C in a C25 incubator shaker (New Brunswick Scientific, Edison, NJ, USA) at 200 rpm. A control culture was grown under the same conditions in the absence of TEB. The resulting mycelium was collected by filtration, frozen in liquid nitrogen, and ground into a fine powder. A total of 300 mg of powdered mycelium were used for protein extraction.

Protein extraction was performed as previously described by Reis et al.^27^ Briefly, 1 mL of extraction buffer was added to the powdered mycelium in microcentrifuge tubes. The buffer consisted of 7 M urea (Cytiva, Uppsala, SE), 2 M thiourea (Cytiva, Amersham, UK), 2% Triton X-100 (GE Healthcare, Piscataway, NJ, USA), 1% dithiothreitol (DTT; Bio-Rad Laboratories, Hercules, CA, USA), and 1 mM phenylmethanesulfonyl fluoride (PMSF; Sigma-Aldrich, St. Louis, MO, USA). The samples were vortexed for 60 min at 4 °C in a refrigerator and then centrifuged at 16,000*g* for 20 min at 4 °C. The supernatants were collected, and protein concentration was determined using the Bradford assay. Absorbance measurements for each sample and a bovine serum albumin (BSA) standard curve (Bio-Rad Laboratories) were performed by using a Synergy 2 Multimode Reader (BioTek Instruments, Winooski, VT, USA) at 485 nm. Before the trypsin digestion step, 100 μg aliquots of protein were precipitated with methanol/chloroform.^28^ The protein pellets were resuspended in 7 M urea/2 M thiourea and digested using the filter-aided sample preparation (FASP) method.^29^ Digestion was carried out by adding 20 μL of trypsin (V5111, Promega, Madison, WI, USA) prepared in 50 mM ammonium bicarbonate at an enzyme-to-protein ratio of 1:100. The resulting peptides were quantified using the A205 nm protein and peptide quantification method on a NanoDrop 2000c spectrophotometer (Thermo Fisher Scientific, Waltham, MA, USA) and then transferred to total recovery vials (Waters).

### LC–MS/MS Analysis

LC–MS/MS analysis was performed using a nanoACQUITY ultraperformance liquid chromatograph (nanoUPCL) system connected to a Q-TOF SYNAPT G2-Si mass instrument (Waters, Manchester, UK) equipped with an electrospray ionization (ESI)-MS/MS source. The analysis included three biological replicates of 2 μg of peptide samples. All mass spectrometry procedures, including instrument settings and configurations, were conducted as previously described by Passamani et al.^30^

Spectral processing and database searches were performed using the ProteinLynx Global SERVER (PLGS) software v.3.0.2 (Waters) according to Passamani et al. (2018). Protein identification was conducted using the *A. fumigatus* protein database from UniProtKB (Proteome ID: UP000002530; https://www.uniprot.org/taxonomy/330879). Label-free quantification analysis was carried out using ISOQuant software v.1.7.^31,32^ For comparative proteomics, only proteins present or absent (for unique proteins) in all three biological replicates were considered for differential accumulation analysis. Proteins with significance according to Student’s *t*-test (two-tailed; *p* < 0.05) were classified as differentially accumulated proteins (DAPs), which were considered up-accumulated if the fold change (FC) was greater than 1.5, and down-accumulated if the FC was less than 0.667. Finally, function annotation was performed using OmicsBox software 3.0.29 (https://www.biobam.com/omicsbox/). The proteomics MS data have been deposited in the ProteomeXchange Consortium via the PRIDE^33^ partner repository with the data set identifier PXD064657.

## Conclusion

Exposure of *A. fumigatus* to the agricultural triazole TEB favored the development of resistance to clinical triazoles, resulting in reduced efficacy of these antifungals widely used in clinical practice. Furthermore, the study demonstrates the microorganism’s adaptation to triazole through maintenance of the ergosterol biosynthesis pathway, activation of EPs, and proteomic alterations.

However, the study has limitations, including the difficulty in fully reproducing the environmental and clinical complexity in which cross-resistance occurs and the lack of genetic sequencing of the target gene of the azole compounds to understand the contribution of specific mutations to the cyp51A gene.

Future studies should be conducted to clarify the occurrence of mutations, especially in the target gene, to elucidate the complete dynamics of resistance. It also highlights the urgency of continuous monitoring of the application of triazole pesticides in the environment to minimize the clinical impact of resistance development.
